# A Novel Adaptation of the HOME Inventory for Elders: The Importance of the Home Environment Across the Life Course

**DOI:** 10.3390/ijerph16162826

**Published:** 2019-08-08

**Authors:** Kathryn Hale, Truls Østbye, Bilesha Perera, Robert Bradley, Joanna Maselko

**Affiliations:** 1 Cecil G. Sheps Center for Health Services Research, University of North Carolina at Chapel Hill, Chapel Hill, NC 27599, USA; 2 Department of Family Medicine and Community Health, Duke University School of Medicine, Durham, NC 27710, USA; 3 Center for Aging Research and Education, Duke-NUS Medical School, Singapore 169857, Singapore; 4 Department of Community Medicine, Faculty of Medicine, University of Ruhuna, Galle 80000, Sri Lanka; 5 Department of Psychology, Arizona State University, Tempe, AZ 85287, USA; 6 Department of Epidemiology, Gillings School of Global Public Health, University of North Carolina at Chapel Hill, Chapel Hill, NC 27599, USA

**Keywords:** healthy aging, developmental life course, mental health, elderly, caregiving arrangements, mixed methods, home environment, Sri Lanka

## Abstract

The context in which dependents, regardless of age, receive care affects their health. This study adapted the Home Observation for Measurement of the Environment (HOME) Inventory, originally designed for child development research, to assess the quality of stimulation and support available to elders in their habitual households in Sri Lanka. Whether the adapted domains correlated with indicators of health and well-being in ways consistent with the child development literature was then examined. Through mixed-methods research based on 248 household surveys, four focus groups, and 15 interviews, three domains emerged: *Physical Environment, Variety of Stimulation,* and *Emotional and Verbal Responsiveness*. Regression modeling revealed that a higher quality physical home environment correlated with two measures of cognitive function after adjusting for covariates, but no consistent association with two psychological well-being scales. In contrast, higher *Variety of Stimulation* scores correlated with better cognitive function and lower psychological distress. There was no consistent correlation between *Responsiveness* and selected health outcomes. Qualitative data indicate that elders are active household contributors who strive to achieve harmonious relations with coresident kin. These findings reveal notable synergies between early and late life efforts to improve cognitive and psychological health, and highlight household considerations for future healthy aging research.

## 1. Introduction

Globally, improvements in healthcare, community infrastructure, and the overall social and economic context have led to higher life expectancy. Scientists have sought to understand human development across the life cycle by giving increased attention to the losses and gains experienced in advanced age, such as increased disease burden [1] and continued emotional and relational growth [2] (p. 2). Despite the high disease burden in elderly populations, there is substantial variability in cognitive functioning throughout the life course, with approximately 50% of older people in high income countries like Germany being able to live independently beyond the age of 90 [3,4]. Furthermore, individuals near the end of the life cycle report greater social satisfaction and less strain from the relationships that they selectively foster and maintain [5]. Most adults over the age of 60 report high subjective quality of life, emotional stability, and life satisfaction [6,7], indicating that decreased psychological well-being may have less to do with the experience of chronological aging than it does with significant life events and losses that occur in older cohorts [8,9,10]. As research on the life course has accumulated, a broader focus on examining elderly individuals who maintain cognitive functioning and social engagement has emerged [11]. In light of these factors, it appears that physical functioning/absence of disability, cognitive functioning, and life satisfaction are the three most consistent components that comprise healthy or successful aging [12] (pp. 9–11).

The focus on successful aging is consistent with a developmental perspective that recognizes how environmental and sociocultural factors influence typical development across the lifespan [13,14]. Irrespective of age, the social and physical environment can be enriched or impoverished in ways that serve as determinants of physical activity, cognitive stimulation, and social support. However, much less research evaluates how elders’ environments and interpersonal relationships combine to support positive mental health and prevent stagnation or isolation. The spectrum of care provided to dependents varies widely in institutional versus family-based settings, ranging from households of biologically related kin to small residential dwellings that care for five to ten unrelated individuals to large institutions such as skilled nursing homes or orphanages. For example, Whetten and colleagues [15] conducted a longitudinal cohort study in this line of research and followed almost 3000 orphans and separated children. They found that children in institution-based care are no worse off than children in family-based care and challenged calls for global deinstitutionalization and implementation of policies supporting household-based care as the gold standard [16] (p. 379). In other words, although the setting of care (institution vs. family) did not predict cognitive development and mental/physical health outcomes, the specific material and social treatment provided in those settings did vary and matter.

A true developmental perspective recognizes the changing needs and capacities of the elderly. On a practical level, elders’ homes may need retrofitting (i.e., repurposing items through the addition of components or accessories that were not present at time of manufacture), and these individuals may need to use assistive devices in the home to support accessibility and to protect themselves from falls or injuries. Elders who live alone or in rural areas may require community assistance to access basic community resources [17,18]. Likewise, if/when elders move into assisted living care, attention needs to be paid to environmental factors (such as pollution), structural conditions (such as living space, usability), and the opportunities for social/cognitive enrichment available to elderly individuals, each of which has been linked to mental and physical health outcomes [19]. Notwithstanding the costs and stress of relocation, many elders desire to “age in place” at home, given that the home can provide a familiar, safe space with community contacts which buffer against loss of personal autonomy [20] (p. 629) and the home environment contains objects imbued with symbolic meaning that contribute to well-being [21,22]. Thus, the quality and predictability of the physical and interpersonal aspects of the home environment often interact with elder health and these factors are incorporated into studies assessing quality of life and successful aging [23,24,25].

As people advance into old age, they often face multiple transitions in their daily lives, including the narrowing of their social networks and range of activities [26] (p. 1381), [19] (p. 234). However, self-reported health and well-being tend to be connected to the individual’s perceived evaluations of their social and physical circumstances [27,28], together with the actual social, economic, cultural and technological context in which they live [29,30,31]. The present study focused on Sri Lanka, a country with one of the most rapidly aging populations in the world [32,33], and sampled households with elders over the age of 60 who lived with either a spouse or, as was most often the case, a child or son-/daughter-in-law who served as a caregiver for the index elderly person. Although the likelihood of living in a nursing facility or assisted living community increases with age in higher income Western societies [34,35], institutional care is very limited in countries like Sri Lanka [36] (pp. 699–700), [37] (pp. 27–28). Furthermore, a national survey found that elderly Sri Lankans living alone were more likely than elders living with a spouse or other family member to have depressive symptoms [38] (p. 234), attributing this living arrangement to serve as a proxy for social isolation and stigmatization. Social participation affords elders protection against a wide range of health problems including heart disease, depression, and dementia [39]. Attentive social networks can have a major impact on maintaining social and cognitive function and improving an elder’s perceived quality of life [19]. Instead, most elders live in their own home or the home of their children [36] (p. 696), [40] (p. 86) and cite traditions and beliefs that adult children have a duty to take care of their elderly parents at home, rather than placing them in assisted living or a nursing home. Thus, there is a need to look carefully at the quality of the social environments experienced by the elderly, and Sri Lanka is a strong setting to empirically assess the geographies of aging within their habitual homes and determine what structures are beneficial to individuals with different histories or needs.

## 2. Purpose of the Study

While experiences connected with the home environment are considered important to the health and well-being of the elderly, there is currently no measure of the overall quality of the home environment that documents the physical and interpersonal conditions of home life that are hypothesized to facilitate healthy aging. In contrast, at the other end of the life course, instruments such as the HOME Inventory (described below) are frequently used to document the quality of the home environment from the perspective of what is most developmentally beneficial to the child and predictive of their later health [41]. HOME Inventory scores are highly predictive of future physical, cognitive, linguistic, and socio-emotional outcomes among children and adolescents [42], and the Inventory has helped inform multiple parenting and child development interventions worldwide (for a review of this work, see Totsika and Sylva [43]). A comparable measure for the elderly population might be similarly beneficial.

We therefore tailored the HOME instrument to systematically assess the physical and social resources available in the home environment of persons 60 and older. We then examined whether this new assessment instrument correlates with measures of cognitive function, psychological distress, and quality of life. We took an explicitly developmental perspective on how the physical and socio-emotional environment influences the aging process (see Thiele and Whelan [13]) and administered the measure to participants with diverse physical and cognitive abilities. We attempted to go beyond the conceptual and empirical literature constructed in Western countries, where much of the research on emotional regulation and stress reactivity in the context of healthy aging has taken place [2] (p. 37). Using mixed methods, we are also able to address issues of physical functioning and meaningfulness of the home to health as well as subjective well-being among the elderly.

## 3. Materials and Methods

The current analysis is based on a cross-sectional survey of 252 community-dwelling elders (aged 60+) and their coresident caregivers, in a peri-urban division (Galle) and three rural divisions within a 50 km radius of Galle in southern Sri Lanka. It is also informed by qualitative data gathered through four focus group discussions (FGDs) held with individuals between 57 and 85 years old in September and October 2011 and fifteen in-depth interviews conducted between August and September 2012. The overall purpose of the larger study was to assess both elder and caregiver challenges associated with declining health, and the present analyses used the survey data and key themes from the qualitative data to assess whether the physical and social resources in the home environment are predictive of better cognitive functioning and psychosocial outcomes.

Eligible households consisted of a coresident elderly person over 60 and an identified caregiver, in either the selected peri-urban or rural divisions. The study sought to conduct the household survey with roughly half the sample in the peri-urban and rural localities. To do so, a household was chosen at random in each division to approach, and interviewers utilized the ‘right hand method’ to continue approaching eligible households. In total, 413 households with an elder over the age of 60 were approached. Forty-seven households were excluded because the elder lived alone, 17 were excluded because the caregiver was not home, and 30 were excluded because the elder had significant health problems. Of the remaining 318 eligible households, 66 refused and one did not complete the interview, resulting in the enrollment of 252 dyads.

Purposive sampling recruited a total of 31 elders between the ages of 57 and 85 to participate in four FGDs that were stratified by gender and urban/rural location. Public Health Midwives from both areas were asked to select 7–8 individuals with good communication skills for each of the four focus groups. Each focus group had a moderator and note-taker that was gender-matched to the group; the moderator used a prepared discussion guide (Appendix A) to guide participants through the discussion while the note-taker wrote notes regarding both verbal and nonverbal content conveyed during the focus groups. Each FGD lasted between 60 and 90 min and sought to understand community norms surrounding aging, forgetfulness, and depression. In addition, 3–5 people from each focus group were asked to identify/refer their caregiver for an in-depth interview (IDI) regarding their caregiving experiences as well as the elder’s mental and physical health status, needs, and family involvement. Five elders from the FGDs and ten caregivers, each of whom as a child or in-law of the referring elder, participated in IDIs which lasted between 30 min and 1 h each. All audio recordings were subsequently transcribed verbatim, and then translated into English by bilingual translators. Two team members who were fluent in Sinhala checked these translations for accuracy and made corrections when necessary.

All participants provided written consent and study procedures were conducted in Sinhala, the local language. The Duke University Health System IRB and the Faculty of Medicine at the University of Ruhuna Ethics Committee provided ethical approval for the study.

### 3.1. Measures

HOME Inventory and its adaptation. The Home Observation for Measurement of the Environment (HOME) inventory is a systematic assessment of the caregiving environment that is traditionally used in the context of childhood development. It involves a 45–90-min home visit with the target participant and their primary caregiver in order to measure the quantity and quality of stimulation and support available to a child in the home environment. (Other household members are welcome to participate, but their presence is not required.) It was originally designed to distinguish environments that pose a risk for children’s development from those environments that provide adequate support for development. It was not designed to discriminate between adequate and highly enriched family environments [44] (p. 253). There are presently five age-specific versions available, as well as a Supplement to the HOME for Impoverished Families [45]. The scale has been widely used outside of the United States, with evidence indicating validity in diverse cultures and family conditions [42,46]. The developers of the inventory posit that the HOME taps social, material, and structural conditions of the family that relate to child development, and that scores correlate with family contextual variables as well as child outcomes in terms of physical, social, and intellectual development [42,44].

In the present study, we modified the Inventory to better reflect our target population, as suggested by one of the Inventory’s developers [47]. In line with our theoretical focus on the life course, we began by reviewing all versions of the HOME inventory and selecting the subscales that were most applicable to and culturally appropriate for an elderly population. We pilot tested the wording to use when referring to our focal population, such as confirming that older adults are referred to as “elders” rather than “seniors” or a role-specific word like “grandparent”. Bilingual investigators engaged in an iterative process to translate, back translate and discuss the resulting product. The entire instrument was piloted and additional refinements were made through consultation with local experts before the start of fieldwork. We identified developmental differences between youth and elders, as well as relevant cultural differences surrounding autonomy vs. family obligation and demographic correlates affecting one’s stance toward individualism vs. collectivism in dyadic interactions [48,49]. We deleted all items contained in the following subscales; modeling, modeling/encouraging maturity, fostering self-sufficiency, and regulatory activities, as they were determined to be more applicable to children than the elderly. We focused on *Variety of Stimulation, Physical Environment,* and *Emotional and Verbal Responsiveness* as cross-cutting domains and either modified the wording of particular items or deleted them because they were deemed inappropriate for elderly individuals (e.g., elder “sees and spends time with father/father figure 4 days a week”) or to Sri Lankan family structure/cultural norms surrounding the expression of warmth and affection (e.g., “primary caregiver caresses, kisses, cuddles, or hugs X once during visit”). This resulted in 29 ‘yes/no’ items, which were primarily based on direct observations in the home. As was the case in the original HOME, when direct observation was not possible, inquiries were made during the 60-min caregiver interview. Items are scored 0 for ‘no’ or 1 for ‘yes,’ with higher scores representing a higher quality environment. Since our data collection period, this instrument has been translated and used with a Sri Lankan sample of mothers and early adolescents [50], but this would be the first instance of using the HOME Inventory in order to assess environmental circumstances that may be connected to cognitive development and psychosocial well-being in an elderly population. Our specific items are provided along with participant response rates in Table 2 on pp. 9-10.

As mentioned above, three HOME domain scores emerged as the most salient in the context of successful aging and were used in our analyses: *Variety of Stimulation, Physical Environment,* and *Emotional and Verbal Responsiveness*. The *Variety of Stimulation* domain taps into the variety of sensory activities and learning stimulation available to the focal participant. It consists of nine items resulting in a score range of 0–9. *Physical Environment* is a 10-item domain evaluating physical aspects of the interior of the home, such as sanitation, safety, crowding, and noise. *Emotional and Verbal Responsiveness* (hereafter referred to as “*Responsiveness*”) is a domain consisting of 10 items that tap into the quality of the communicative and affective interactions between the caregiver and the focal participant. Raters used the entire range of possible scores when administering the adapted version of HOME, with scores ranging from 1 to 10 on *Physical Environment*, 1 to 10 on *Responsiveness*, and 0 to 9 on *Variety of Stimulation*. As part of the analysis, we constructed three groupings of participants on each of the HOME domains by splitting scores into tertiles.

Cognitive Function

To assess cognitive function as it relates to the home environment, we relied on the elder administered Montreal Cognitive Assessment (MoCA) [51]. We also used the Informant Questionnaire on Cognitive Decline in the Elderly (IQCODE) as administered to the coresident caregiver [52]. We chose to utilize both of these measures because they offer two complementary perspectives on cognitive function: the MoCA provides an objective assessment of current cognitive functioning while the IQCODE elicits observed changes during the last 10 years.

MoCA. The MoCA assesses 11 domains including visuo-spatial, executive function, short-term memory, attention, concentration, working memory, language, and orientation [51]. This objective test has been previously used internationally in community surveys [51,53,54], and in Sri Lanka in particular [55]. The MoCA was developed as a tool to screen patients with mild cognitive complaints who usually perform in the normal range on the Mini Mental Status Exam [56]. MoCA scores range from zero to 30, with higher scores indicating better cognitive function, and scores below 24 indicating possible dementia in Sri Lanka, per the Sinhala validation study [55] (p. 150). As done by the scale’s creators [51] (p. 697), one point was added to the total MoCA score for participants with ≤12 years of education (if total MoCA score was <30). This was the case for over two-thirds of the elders in our study sample.

IQCODE. The IQCODE consists of 21 questions posed to the caregiver. Its purpose is to assess changes in an elder’s cognitive functions during the last 10 years [52,57]. In response to queries about daily tasks such as misplacing household items, the caregiver states whether this has “much improved,” “not changed much,” or become “much worse” (scored on a scale of 1–5, respectively) over the last 10 years. The responses are averaged and higher values are indicative of greater decline (ibid), such that the measure can be used as a screening test for dementia. These mean values can also be grouped so that scores below 3.0 indicate improvement, scores of 3.01–3.50 indicate slight decline, scores of 3.51–4.0 indicate moderate decline, and scores above 4 are indicative of severe decline. (Quinn and colleagues [58] provide a review of IQCODE cut-points and the effects of those thresholds on sensitivity and specificity.) This scale can be used in all patients irrespective of their present physical condition or level of education. Researchers have identified the IQCODE as more effective and culturally acceptable for use as a screening tool for dementia in Sri Lanka when compared with the Mini Mental Status Exam (MMSE) and the Clinical Dementia Rating (CDR) after translating, culturally adapting, and validating the scales in Sri Lanka [59].

Psychosocial Outcomes

The two main measures of psychosocial outcomes were the Kessler Psychological Distress Scale (Kessler-10) and the Index of Capability for Older Adults (ICECAP-O). We included both of these measures in order to document self-reported psychological distress and quality of life or meaning-making alongside objective measures of health and housing characteristics.

Kessler-10. The Kessler Psychological Distress Scale (Kessler-10) is a 10-item questionnaire that yields a global measure of distress based on symptoms of depression and anxiety experienced during the past 30 days [60]. Questions screening for feelings of fatigue or restlessness are rated on a 5-point Likert frequency from “none of the time” to “all the time” (scored 1–5, respectively, with a total score ranging from 10 to 50). This scale has been used in multiple countries [61,62], and it has been translated to Sinhala and validated in a Sri Lankan population [63]. Scores above 24 indicate a moderate or severe mental disorder.

ICECAP-O. The Index of Capability for Older Adults [64] is a brief interview measure for elders aged 65+ that assesses not only health-related quality of life, but also how a person’s disabilities affect his or her perceived ability to perform activities of daily living (ADL) within their present economic and environmental conditions. The ICECAP-O links capability with age and well-being [64,65,66,67] and provides information about accessibility and perceived barriers to “freedom”, both in relation to not having to worry or work in order to meet their own needs and toward having the personal autonomy to participate in social, economic, or religious activities, which previous research has identified to be very important to this population [68] (pp. 847–849). The ICECAP-O scale was developed in the UK and has been applied internationally [69] (p. 89), although not previously in Sri Lanka. The measure includes five questions regarding an individual’s ability to pursue different attributes of quality of life including (1) attachment (assessed through a question about love/friendship), (2) security (concern about the future), (3) experiencing enjoyment, (4) role (feeling valued), and (5) control (behavioral independence). Responses on individual items range from 1 (little ability) to 4 (great ability) to pursue the things found to be important in life, and they are summed with a total score ranging from 5 to 20.

### 3.2. Analytical Strategy

The authors used a mixed methods approach that embeds one method (i.e., qualitative) within another (i.e., quantitative) in order to evaluate the adapted HOME Inventory [70]. In the present study, we drew from the FGDs and IDIs to contextualize the quantitative results and provide evidence regarding the manner in which elders engaged with their home environment. To do this, the first author reviewed the qualitative transcripts before conducting quantitative analyses and constructed analytical codes from emergent themes using grounded theory [71]. Analytical codes were grouped and assessed for frequency within the dataset, and then axial coding sought to characterize the conditions and consequences of codes relevant to the elder’s home life. Several themes characterized the elders’ daily activities in the home, and they are discussed below.

Subsequently, in order to examine relations between demographic variables and the HOME Inventory collected during household surveys, we conducted *t*-tests and χ^2^ tests as appropriate. We then used ordinary least squares (OLS) regression models to examine the association between each of the three domains of the tailored HOME (*Variety of Stimulation, Physical Environment,* and *Responsiveness)* and measures of cognitive function as well as psychological distress/well-being (StataSE 15.0). Statistical analyses included all individuals with no more than one missing value on the HOME instrument, yielding a total analytic sample of 248 of our 252 households. Our results report beta coefficients and standard errors corresponding to a point increase in the MoCA, IQCODE, Kessler-10, or ICECAP-O score between the low, medium, or high categories in a given HOME domain. Multivariable regression models were used to assess the impact of HOME Inventory scores on measures of cognitive and psychosocial outcomes. All models were adjusted for age, gender, education (categorized as “never schooled”, “grades 1–5 of primary school”, “grades 6–10 of secondary school”, or “more than secondary school”), and marital status (married/not married).

## 4. Results

### 4.1. Sample Description

The 248 individuals included in our analyses had a mean age of 71.8 (range: 58–92), and over 22% of the sample were above the age of 80, thereby falling into the category of “old-old age” when compared to other studies conducted in Sri Lanka [38,72,73]. One-hundred-and-five participants (42.3%) lived in an urban area and the remaining 57.7% lived in a peri-urban area. The majority of our sample was married (71%), 67% were female, and over 91% identified as Sinhalese. The remainder identified as either Tamil (5.7%) or Muslim (2.8%). Two thirds of our sample (67%) had a 10th-grade education or less. Despite one-third of the sample displaying significant cognitive problems/decline and over half (64.9%) reporting a serious physical illness (such as arthritis or high blood pressure), elders in our study were mostly mobile and self-sufficient and reported a high quality of life. Twenty-three percent of participating elders were employed and engaged in economic pursuits, and another 27% identified as a housewife or student. Many elders sought to contribute to the household or their community in meaningful ways, such as volunteering to organize community sporting events through Elder Societies, participating in governmental meetings (36.7%), or caring for their grandchildren on a daily basis (62.9%) [74].

The mean cognitive function score as assessed by the MoCA was 18.7, and 71% were considered cognitively impaired with a score of 24 or less. This high level of impairment is consistent with the relatively low educational attainment of sample participants. Most caregivers (57%) reported that the elderly person had, on average, slightly declined in cognitive function over the last 10 years. Less than a quarter (23%) of elders had declined to a moderate or severe extent. The mean IQCODE score was 3.3, and 32% of elders qualified for dementia based on a cutoff score of 3.38. Both measures were correlated with age (r = 0.37 for MoCA and 0.36 for IQCODE) and women had slightly lower cognitive function scores (MOCA = 17.8 women, 20.6 men) and were more likely to be perceived as declining by their caregivers (38.8% women were judged to be declining vs. 19.5% men).

With respect to our psychological outcomes, the study sample had a mean score of 16.6 on the Kessler-10 scale for psychological distress, which signaled they were likely to be well. Twenty-six participants (or 10% of our study sample) received a score of 25 or higher, reflecting moderate to severe psychological distress during the last 30 days. The mean quality of life/functioning score as assessed by the ICECAP-O was 17.4, indicating that our sample felt they had a strong capability to “do” and “be” the things that were important to them in life. Two-thirds of the sample had a high score ranging between 16 and 20 points total. Sample characteristics are presented in Table 1.

### 4.2. HOME Inventory Results

Table 2 presents the individual HOME items and the percentage of households given credit for each item. In the *Physical Environment* domain, there was a wide range of responses on individual items. For example, only 21.8% of households were well lit and not perceptually monotonous, whereas 88.3% of households were clean and minimally cluttered. The overall mean for the *Physical Environment* domain was 6.78 (SD = 1.9). Households with *Physical Environment* domain scores of 1–5 (*n* = 56) were categorized as ‘low’, households with scores of 6–7 (*n* = 85) were categorized as ‘moderate’, and households with scores of 8–10 (*n* = 107) were categorized as ‘high’.

Although the *Variety of Stimulation* domain had an overall mean of 3.87 (SD = 1.9) and very few households scored highly, it still displayed wide item-level variability. For example, only 17.7% of participating households had taken an elder to a live musical event or theater performance within the past year, but 80.2% of households reported that elders see friends and other relatives on a regular basis. Households with scores of 0–2 (*n* = 62) were categorized as ‘low,’ households with scores of 3–4 (*n* = 99) were categorized as ‘moderate,’ and households with scores of 5–9 (*n* = 87) were categorized as ‘high.’ In the *Emotional and Verbal Responsiveness* domain, the least common behavior was seeing the caregiver talk with the elder twice during the home visit, occurring in only 38.7% of households. This may be an artifact of the study design, though, as both the elder and caregiver were interviewed at the same time, leaving less opportunity to document naturally occurring interactions. In this domain, the most commonly observed behaviors reflected the presence of clear communication between the caregiver and the interviewer (e.g., Items #26 and #29 below). Households with scores of 1–6 (*n* = 93) were categorized as ‘low’, households with scores of 7–8 (*n* = 71) were categorized as ‘moderate’, and households with scores of 9–10 (*n* = 84) were categorized as ‘high.’ The *Responsiveness* domain had a mean of 7.04 (SD = 2.3) and was strongly skewed to the left, with over half of households receiving a score of 7–10. This is examined in more detail in the Discussion section.

Overall, households with elders who were more educated and younger had higher scores on the HOME domains, while neither gender nor marital status was correlated with HOME scores (analyses not shown). Next, we examined the association between each of the HOME domains and indicators of cognitive function and psychological health (Table 3). After adjusting for potential confounding variables in this population, an individual scoring high in the *Physical Environment* domain had a MoCA score 2.06 points higher than someone in the low reference group (CI = 0.57, 3.56), indicating a lower level of cognitive impairment. Likewise, an individual scoring high in the *Variety of Stimulation* domain had a MoCA score 1.70 points higher than someone in the reference group (CI = 0.18, 3.22). The *Responsiveness* domain was not significantly related to MoCA scores.

Echoing our findings with the MoCA, an individual scoring high in the *Physical Environment* domain had an IQCODE score 3.88 points lower than the reference group (CI = −6.77, 0.99), indicating lower rates of cognitive decline. Neither *Stimulation* nor *Responsiveness* was significantly related to IQCODE scores.

Psychological distress was only predicted by *Variety of Stimulation*, with those scoring high in that domain having a Kessler-10 score 3.15 points lower than those in the low stimulation group (CI = −5.24, −1.05). Neither the *Physical Environment* nor *Responsiveness* domains were significantly related to the Kessler-10. Conversely, the ICECAP-O measure of quality of life was positively correlated with high levels of all three HOME domains.

The ICECAP-O had stepwise changes in effect, where individuals scoring high in any of the HOME domains were consistently more likely to experience a significant improvement in their quality of life. An individual scoring high on the *Physical Environment* domain had an ICECAP-O score 0.92 points higher than the reference group (CI = 0.14, 1.70), indicating a higher ability to pursue different attributes contributing to their quality of life. An individual scoring high on the *Variety of Stimulation* domain had a score 1.29 points higher than the reference group (CI = 0.51, 2.06), and someone scoring higher on the *Responsiveness* domain had a score 0.74 points higher than the reference group (CI = 0.003, 1.47).

### 4.3. Integration with Qualitative Results

The qualitative data provided additional insight into the interpretation of our study results and indicated that our participants were actively engaged with their home environment. Elders sought stimulation and engagement on their own terms, whether it was through household chores, family activities, or community service. This is consistent with our findings that a more stimulating environment is correlated with both higher cognitive function and better psychological health, but it clarifies that the elders were active creators rather than passive recipients of such experiences.

Despite a large portion of our sample having physical and cognitive impairments, high ICECAP-O scores indicated that elders felt capable of performing activities of daily living within their current economic and environmental constraints. Confirming this fact, almost half (46.7%) of interviewees woke as early as 5 am and either worked the fields (most often cinnamon or tea plantations) or helped with childcare and chores around the house to the extent they were able. Some male elders who did not help with household chores served in leadership roles in the community instead. One caregiver explained that to live a happy life, her father “Gets up early, sweeps the garden, sweeps the house. Getting some exercise is good for the health, rather than just sitting there doing nothing.” Although many elders’ adult children encouraged them to relax and simply enjoy retirement, the elders explained that after working hard for much of their lives, they had become so accustomed to it that they would not know what to do if they stopped.

Elders sought to engage in activities that were meaningful for themselves or for their family, including caring for household pets or having the freedom to contribute to household finances without being expected or pressured to do so. One man in the rural FGD explained that in order to age well “without troubling anybody in the society … [we should] earn our own income and use it to have a balanced life.” Talk of balance and unity permeated participants’ dialogue of family life, personal health, and financial or social difficulties, and was likely related to Ayurvedic concepts of imbalance leading to illness. Frequently, caregivers cited family disputes and economic difficulties as a major barrier to giving their elderly parent an ideal life. One interviewee explicitly said that anger and other strong emotions were unhealthy and could cause depression or sadness. The primary barrier to aging well was “… the absence of harmony and peace among the family members and the society, especially with the children. Ah, and economic difficulties can be another reason.” Elders described becoming more likely to lose their temper when the family was not living in harmony, and relatives said it was very difficult to live with people like that—sometimes going as far as stating that those instances were likely to be when “children put their parents in ‘old people’s homes.’”

Elders voiced strong feelings of family responsibility and fretted over ever becoming a burden to their adult children. Two interviewees and members of one focus group explained that their ability to stay healthy and relatively independent from needing intensive assistance would dictate how long they hoped to live. One female focus group participant said, “I would want to ask God to shorten my life … I don’t want to be a nuisance to my children anymore.” When asked how he feels about getting older, another elder replied, “I do not feel that I have grown old yet (smiling). I see such a person as one who has lost everything, become disabled … and left to solitude.” For him and for others, the process of chronological aging was not synonymous with senescence, and individuals looked forward to life for as long as they could contribute to the household, develop their spiritual practice, and maintain a positive quality of life, revealing variable developmental trajectories in old age that speak directly to our study interests.

Our qualitative data also underscored a strong ethos of accepting one’s circumstances without complaint and fostering balance and gratitude in the household, anchored in the Buddhist tradition with which almost 92% of our sample identified. This was illustrated by one grandmother who would run to the toilet and cry whenever she received news of a death or quarrel in the area because she did not want to bother anyone else with her grief. To live happily and remain healthy, household “unity” or “living in harmony” was paramount. Informed by Buddhist beliefs that self-sacrifice and religious practice lead to spiritual enlightenment and positive social standing, half (7/15 or 47%) of participating caregivers responded to questions regarding their elderly parent’s health or happiness with an emphasis on achieving peace and harmony and treating one another properly. When asked if there are any parts of caregiving that they dislike, such as when a parent gets angry, one daughter stated, “She never gets angry,” following up with a smile. These cultural norms inform our understanding of how closely elder health status and the interpersonal home environment are connected in local participants’ perspectives, in addition to raising a possible explanation for the lack of variability found in the *Responsiveness* domain.

## 5. Discussion

Overall, our findings suggest that a more enriched physical and interpersonal home environment was associated with higher cognitive function, lower psychological distress, and better quality of life. Central findings include a significant positive relationship (1) between the cleanliness, safety, and noise of the home’s physical environment and higher cognitive function, as measured by an objective assessment (MoCA) and an informant reported (IQCODE) measure of cognitive function, as well as (2) between the variety of activities and visual/social stimulation available to the elder in the home and both higher cognitive functioning as well as lower levels of psychological distress. There was only one significant finding for the relationship between social responsiveness and quality of life, as measured by the ICECAP-O.

Our combined use of quantitative and qualitative analysis provides a methodological contribution to better strengthen our understanding of the HOME Inventory and how to iteratively adapt it for use with older adults. It also underscores the level of agency and active engagement with the physical and interpersonal environment that elders displayed, distinguishing them from other developmental stages (e.g., infancy) where individuals more passively absorb their environment. By remaining engaged in meaningful activities and feeling capable of achieving their desires (with the ICECAP-O serving as a proxy measure of those sentiments), elders actively engaged with their environment and found stimulation and satisfaction in their achievements as they grew older. This is significant given past research by Siddhisena [75] citing an inability to participate in the home as one of three top reasons for elders to enter an institutional care setting. The original HOME instrument was designed to capture interdependent aspects of the home environment which support children’s developmental outcomes, and findings from this study indicate that some of the same types of physical and social resources in the home environment can improve their capacity to achieve the desires so many reported. Also of note is that each HOME domain is comprised of several subcomponents (such as *Variety of Stimulation* including pet ownership and belonging to a club). Although our study was not designed to examine the impact of specific items, the constructs they reflect may each make a unique contribution to health. Even so, while it is useful to think about the impacts of particular forms of stimulation and different categories of stimulation, having experience with more different types of stimulation over time tends to support all areas of development because humans as a species engage with and transform the environments they inhabit. In sum, this mixed-methods work strengthens our understanding of the diversity which exists in the elderly stratum of the world’s population [76] (pp. 17–21) and echoes calls for meaning-based models of balanced or harmonious aging [77,78].

There was a strong correlation between the *Variety of Stimulation* items of the HOME instrument and performance in cognitive function and psychological health, as measured by the MoCA and the Kessler-10, even after adjusting for demographic factors. This instrument taps into the variety of activities and learning stimulation available to the focal participant, as shown by it reproducing the substantial correlation of the HOME with cognitive measures for children in a new population [42], [43] (pp. 27–28). Specific items from the *Physical Environment* and *Variety of Stimulation* domain ask about the presence of pictures on the wall and the elder’s participation in outdoor spaces and activities, and these items not only speak to benefitting cognition by providing stimulation, but they also comprise part of what makes a house into a meaningful home for elders. After all, photos are likely to be pictures of family or gifts given by relatives or close friends [25]. Our findings also fit well with previous research, showing that older adults with robust social networks and frequent social interaction are less likely to experience decline in cognitive functioning [79,80] and more likely to benefit from their protective function for mental health [81,82]. The potential exists to use cognitive exercises or social engagement to slow cognitive decline [83].

The *Responsiveness* HOME domain had the weakest correlations with measures of psychological distress and quality of life despite our hypothesis that psychosocial scales would correlate with observed communication and psychosocial support between caregiver and elder. However, this appears to mirror a general finding of *Responsiveness* showing the weakest correlation with cognitive development or other measures of child competence [44] (pp. 254–255). Researchers in North America and elsewhere throughout the world have found that HOME items measuring learning stimulation were more highly correlated with children’s cognitive development than the domain tapping socio-emotional support [84,85]. When interpreting the meaning of those findings, it is important to note that there were few very low scores on *Responsiveness*. The cultural context in Sri Lanka also raises the possibility that local cultural models for child socialization might diverge from that of Western countries and teach people to want less and instead be attuned to others’ unspoken needs and affective stance. As a result, nonverbal displays of closeness and care may be more commonly emphasized (see Bradley & Corwyn [46] and Chapin [86] (pp. 69–111)). If so, then the items comprising the *Responsiveness* domain may not sufficiently cover the ways to express verbal and emotional connection to the elderly in Sri Lanka. Future research should keep in mind that broad functions, like responsiveness, may be accomplished using different forms in different contexts or different relationship roles [87]. Certain verbal or behavioral interactions may be more telling in one setting than they are in another, such that the absence of negative exchanges with caregivers may be more consequential for the elderly than the presence of positive exchanges [88].

Furthermore, the home environment is also likely influenced by a number of local factors external to the household, such as urbanicity and other community-based resources which we were not able to address in this study. Although our models adjust for education and home ownership by either the elder or by his/her children was nearly universal, residual confounding by socioeconomic status remains possible. Finally, cultural norms and social desirability strongly influence what is revealed to an interviewer. Our qualitative data underscored a strong ethos of accepting one’s circumstances without complaint and fostering balancing and gratitude in the household. Individuals suppressed complaints, and instead favored avoidance and internal regulation, and were generally hesitant to speak poorly of others or identify conflicts/shortcomings in eldercare, which may have led participant to underreport psychological distress and should be addressed when tailoring this instrument in the future.

## 6. Conclusions

This was the first adaptation of an instrument like the HOME for use across the life course. The household environment is extremely important to Sri Lankan elders who are cared for at home by their kin, and our tailored version of the HOME instrument was successful in predicting developmental outcomes and health states in an aging population. This novel study extends the developmental logic of the HOME instrument across the lifespan and our findings indicate that a more enriched physical environment and a more interactive interpersonal environment in the home are associated with higher cognitive function, lower psychological distress, and better quality of life. In light of recent petitions by groups such as the Human Rights Commission of Sri Lanka [89] for development and intervention plans around elder care, we call for future research regarding the home life and interpersonal relationships of elders and interventions that may positively influence mental health and life satisfaction in old age.

## Figures and Tables

**Table 1:** Characteristics of study participants (*n* = 248).

Category	Number (Percent)	Mean (SD)	Range
**Gender**			
Female	167 (67%)		
**Age**		71.6 (8.2)	57–92
60–64	62 (25%)		
65–69	51 (21%)		
70–74	45 (18%)		
75–79	35 (14%)		
≥80	55 (22%)		
**Education**			
Never schooled	12 (5%)		
Primary (grade 1–5)	72 (29%)		
Secondary (grade 6–10)	82 (33%)		
Passed GCE (O/L)	52 (21%)		
Passed GCE (A/L)	22 (9%)		
Graduate/Post-Graduate	8 (3%)		
**HOME Inventory**			
Physical Environment		6.78 (1.9)	1–10
Variety of Stimulation		3.87 (1.9)	0–9
Responsiveness		7.03 (2.3)	1–10
**Cognitive Function**			
MoCA ^a^		18.7 (6.4)	4–30
Impaired (<24)	177 (71%)		
Unimpaired (≥24)	71 (29%)		
IQCODE ^b^		3.3 (0.5)	1.5–5.0
Dementia (≥3.38)	80 (32%)		
No dementia (<3.38)	168 (68%)		
**Psychosocial Outcomes**			
Distress (Kessler-10) ^c^		16.6 (6.5)	10–43
Quality of Life (ICECAP-O) ^d^		17.4 (2.4)	5–20

^a^ MoCA: Montreal Cognitive Assessment; ^b^ IQCODE: Informant Questionnaire on Cognitive Decline in the Elderly; ^c^ Kessler-10: Kessler Psychological Distress Scale; ^d^ ICECAP-O: Index of Capability for Older Adults.

**Table 2:** Affirmative responses to Home Observation for Measurement of the Environment (HOME) items.

Physical Environment	Percent “YES“
1. House or apartment is free of potentially dangerous structural or health hazards —e.g., stairs with no railings, unmanaged wastage, slippery floor	75.8%
2. Is the home clean; all visible rooms are reasonably clean and minimally cluttered	88.3%
3. Home has at least 100 square feet of living space per person	81.5%
4. In terms of available floor space, the rooms are not overcrowded with furniture	47.6%
5. The interior of the house or apartment is not dark or perceptually monotonous	21.8%
6. House or apartment has at least 2 pictures or other types of artwork on the walls	64.5%
7. House or apartment is not overly noisy—from noise inside the home—e.g., television, shouting, radio	83.9%
8. House or apartment is not overly noisy—from noise outside the home—e.g., traffic, people, music	65.3%
9. Household members do not use tobacco	67.7%
10. There are no obvious signs of recent alcohol use (beer or liquor bottles)	81.5%
**Variety of Stimulation**	
11. Home has a pet	55.6%
12. Does elder see friends and other relatives regularly?	80.2%
13. Elder eats one meal per day, on most days, with caregiver and other household members.	71.7%
14. Does elder do any outdoor activities with any family members? (E.g.: Going to the park)	35.1%
15. Does elder go on outings with any family members at least once a month?	45.2%
16. Has elder gone to any cultural, artistic, or historic exhibit or event (not counting religious festivals) in the last year?	22.2%
17. Has family member taken elder or arranged for elder to attend some type of live musical or any type of theater performance within the past year?	17.7%
18. Does elder belong to any clubs or organizations or take any kind of lessons?	32.6%
19. Does elder participate in activities/hobbies regularly with caregiver or other family members?	26.6%
**Emotional and Verbal Responsiveness**	
20. Caregiver talks with elder twice during visit (beyond introduction and correction)	38.7%
21. Caregiver encourages elder to contribute to the conversation during visit by getting him/her to relate an experience OR by taking time to listen to him/her relate an experience	50.4%
22. Caregiver mentions particular skill, strength, or accomplishment of elder during interview during visit	50.8%
23. Caregiver spontaneously praises elder’s qualities or behavior twice during visit	55.2%
24. When speaking of or to elder, caregiver’s voice conveys positive feelings	90.3%
25. Caregiver shows some positive emotional response to praise of elder offered by interviewer	83.9%
26. Caregiver’s speech is distinct, clear, and audible to the interviewer	97.2%
27. Caregiver initiates verbal interchanges with the interviewer, asks questions, makes spontaneous comments	75.0%
28. Caregiver expresses ideas freely and easily and uses statements of appropriate length for conversation	66.1%
29. Caregiver appears to readily understand the interviewer’s questions	95.9%

**Table 3:** Association between the three HOME domains and indicators of cognitive function and psychological distress ^1,2^.

HOME Domain		MoCA (Higher Score = Less Impairment)		IQCODE (Higher Average = Cognitive Decline)		Kessler-10 (Higher Score = Higher Distress)		ICECAP-O (Higher Score = Higher Capability)	
		β (95% CI)	*p*-value	β (95% CI)	*p*-value	β (95% CI)	*p*-value	β (95% CI)	*p*-value
Physical Environment	Reference								
	Moderate	1.01 (−0.53 to −2.55)	0.20	−3.96 (−6.95 to −0.97)	0.01	−0.23 (−2.42 to 1.96)	0.84	0.48 (−0.32 to 1.29)	0.24
	High	2.06 (0.57 to 3.56)	0.01	−3.88 (−6.77 to 0.99)	0.01	−0.99 (−3.10 to 1.13)	0.36	0.92 (0.14 to 1.70)	0.02
Stimulation	Reference								
	Moderate	1.61 (0.15 to 3.07)	0.03	−0.29 (−3.15 to 2.60)	0.84	−3.31 (−5.33 to −1.29)	0.001	0.28 (−0.47 to 1.02)	0.47
	High	1.70 (0.18 to 3.22)	0.03	−2.12 (−5.09 to 0.85)	0.16	−3.15 (−5.24 to −1.05)	0.003	1.29 (0.51 to 2.06)	0.001
Responsiveness	Reference								
	Moderate	−0.27 (−1.70 to 1.17)	0.72	0.58 (−2.21 to 3.37)	0.68	1.03 (−0.97 to 3.03)	0.31	0.45 (−0.29 to 1.19)	0.23
	High	−0.24 (−1.67 to 1.18)	0.74	0.06 (−2.71 to 2.83)	0.97	−0.64 (−2.62 to 1.35)	0.53	0.74 (0.00 to 1.47)	0.05

^1^ linear regression model adjusted for age, gender, education, and marital status. ^2^ four deleted observations/participants (*n* = 248). Note: Education was analytically grouped as follows: never schooled, primary, secondary, or more than secondary.

## Appendix A. Focus Group Discussion (FGD) Guide

**Number of FGDs:** 4 total (2 urban and 2 rural, segregated by gender)

**Composition of each FGD:** 6–8 elderly individuals

**I. Demographics**

Prior to starting the FGD, participants will be asked to complete a brief demographic sheet that includes information about age, marital status, household composition, etc.

**II. Aging**

We would like to hear your opinions about what it is like to be an older person in Sri Lanka.

- Could you describe the role of older people in Sri Lanka?
- What are some of the good things of growing older?
- What are some of the bad things of growing older?
- What is it like to grow older? What experiences do you have that you did not have during your younger days? (Seek personal examples.)

Let us talk about what a good life looks like for an older person.

Think about an ideal life for an older person, for example for an 80 year old man/woman.

- What does this life look like?
- Where does this person live?
- Who takes care of him/her?
- What does he/she do all day?
- Tell me about older people whose lives you admire. What are they like? Why do you admire their lives?
- What are things that older people can *do* that helps them to live a good and healthy life?
- What are some barriers to achieving this ideal life? (Moderator should probe for social, structural, economic barriers.)

**III. Problems and Coping**

Please tell me about the types of problems older experience in their lives.

When people experience these problems, what do they do to address/manage them?

- Probe for ways they deal with specific problems they just mentioned.
- How do older people use religion and religious practices to deal with their problems?

**IV. Mental Health**

I would like to hear your thoughts about “depression”.

How can this be described in Sinhalese?

- What is depression?
- How can you tell that someone is depressed? What does it look like? (probe for behaviors and physical symptoms)

Let’s talk about depression among older people.

- Do people get more or less depressed as they get older? Why is that?
- What might be reasons that older people experience depression?
- Have you known an older person who has been depressed? What did this look like?
- What should the family do when an older person starts to behave/feel/think this way, if anything?
- How are older people who are depressed treated? Are they treated differently by others (family and community members) because of their depression? Why are they treated this way?
- Do families hide the fact that an older family member is depressed? If so, why?
- We know that people sometimes get more forgetful as they get older.
- What types of problems have you seen related to forgetfulness?
- When, if ever, is it necessary to go to a doctor? (Moderator should probe whether this depends on age, severity of forgetfulness.)
- Describe a person who you think should be taken to a doctor due to forgetfulness?
- What other places might families go to get help for forgetfulness?

**V. Caregiving**

As people get older, they often need help from other people in order to take care of their basic needs. Let us talk about this.

- What type of help and support do people need as they get older? (Moderator should generate a comprehensive list of needs.)
- How do different family members provide this help and support to older people?
- Who usually holds the main responsibility to help the elderly to meet these needs? Why do you say this?
- What makes a good caregiver? What is an ideal situation and type of person to take care of an older person?

I would also like to hear about what the older person can give back to the family.

- What types of support can the older family member give to others?
- How can the elderly contribute to their families?

**VI. Leisure activities**

Let us talk about what you do in your free time.

- How do you spend your free time? (Moderator should generate a list of activities and get a sense of what is understood by the Sinhalese word for “leisure”.)
- Are there things you would like to do in your free time that you are not able to do? Why?

**VII. Conclusions**

We have talked about what it is like to be an older person, issues of mental well-being, and what you would like and expect from family members who might help to take care of you. Is there anything else you would like to add?
